# Rapid detection of phenotypes Bombay *se*^*del*^ and nonsecretor rs200157007 SNP (302C > T) by real-time PCR-based methods

**DOI:** 10.1038/s41598-021-94659-7

**Published:** 2021-07-22

**Authors:** Mikiko Soejima, Yoshiro Koda

**Affiliations:** Department of Forensic Medicine, Kurume University School of Medicine, Kurume, 830-0011 Japan

**Keywords:** Genetics, Molecular biology, Molecular medicine

## Abstract

The *se*^*del*^ allele is one of the nonsecretor alleles (*se*) of *FUT2* generated by an Alu-mediated recombination event and was first found in Indian Bombay phenotype individuals who have anti-H, anti-A, and anti-B antibodies in their serum. As well as anti-A, and anti-B antibodies, anti-H is clinically significant because it causes sever hemolytic transfusion reactions. Like *se*^*del*^, *se*^*302*^ having a missense single nucleotide polymorphism (SNP), 302C > T, is characteristic of South Asians with a frequency of 10–30%. We developed a real-time PCR melting curve analysis for detection of *se*^*del*^ using a 127-bp amplicon encompassing the breakpoint junction. In addition, by performing duplex PCR by amplifying a 65-bp amplicon of the *FUT2* coding region at the same time, we could determine the zygosity of *se*^*del*^ in a single tube. We also developed an Eprobe-mediated PCR assay (Eprobe-PCR) for detection of 302C > T of *FUT2.* These methods were validated by analyzing 58 Tamils and 54 Sinhalese in Sri Lanka. Both the duplex PCR melting curve analysis for determination of *se*^*del*^ zygosity and the Eprobe-PCR assay for detection of 302C > T exactly determined three genotypes. In addition, the results of the present methods were in complete agreement with those obtained by previously established methods. The two present methods were reliable and seem to be advantageous for large-scale association studies of *FUT2* polymorphisms in South Asian populations.

## Introduction

In humans, expression of the H antigen, a precursor of A and B antigens of the ABO blood group, is regulated by two α(1,2)fucosyltransferases. One is the H enzyme encoded by *FUT1*, which regulates expression of the H antigen, and thereafter ABO antigens, on the cell surface of erythrocytes and endothelial cells. The other is the Se enzyme, encoded by *FUT2*, which regulates expression of the H antigen on mucosal surfaces and in body fluids^1,2^. Deficiency of the H enzyme (Bombay and para-Bombay phenotypes) is very rare, whereas deficiency of the Se enzyme (nonsecretor) is common^1^. The Bombay phenotype individuals do not express H antigen and hence A antigen or B antigen either on red cells or in secretions, because they lack both H and Se enzyme activities. As a result, they have anti-H, anti-A, and anti-B antibodies in their serum and can receive only autologous blood or blood from another Bombay phenotype individual. Transfusing blood group O red cells to them can cause severe hemolytic transfusion reactions^1^. Functional alleles of *FUT1* (*H*) and *FUT2* (*Se*) are dominant over nonfunctional alleles of *FUT1* (*h*) and *FUT2* (*se*)^1^. *FUT1* and *FUT2* locate on chromosome 19q3.3 beside a pseudogene (*SEC1*) having high sequence similarity particularly to *FUT2*^3^.

Several single nucleotide polymorphisms (SNPs) of *se* alleles are distributed in a population-specific manner^4–6^. The 428G > A nonsense SNP (rs601338, W143X) constituting *se*^*428*^ is common in Europeans, Africans, and West Asians with a frequency about 50% and in South Asians with a frequency of 10–30%. On the other hand, the 385A > T missense SNP (rs1047781, I129F) constituting a weak secretor allele (*Se*^*w*^) is common in East and Southeast Asians with a frequency about 50%. In addition, the 302C > T missense SNP (rs200157007, I101P) constituting *se*^*302*^ was first identified in a Thai population with a low frequency and was demonstrated to be exclusively encountered in South Asians with a frequency of 10–30%^5,7,8^.

**Figure 1:**
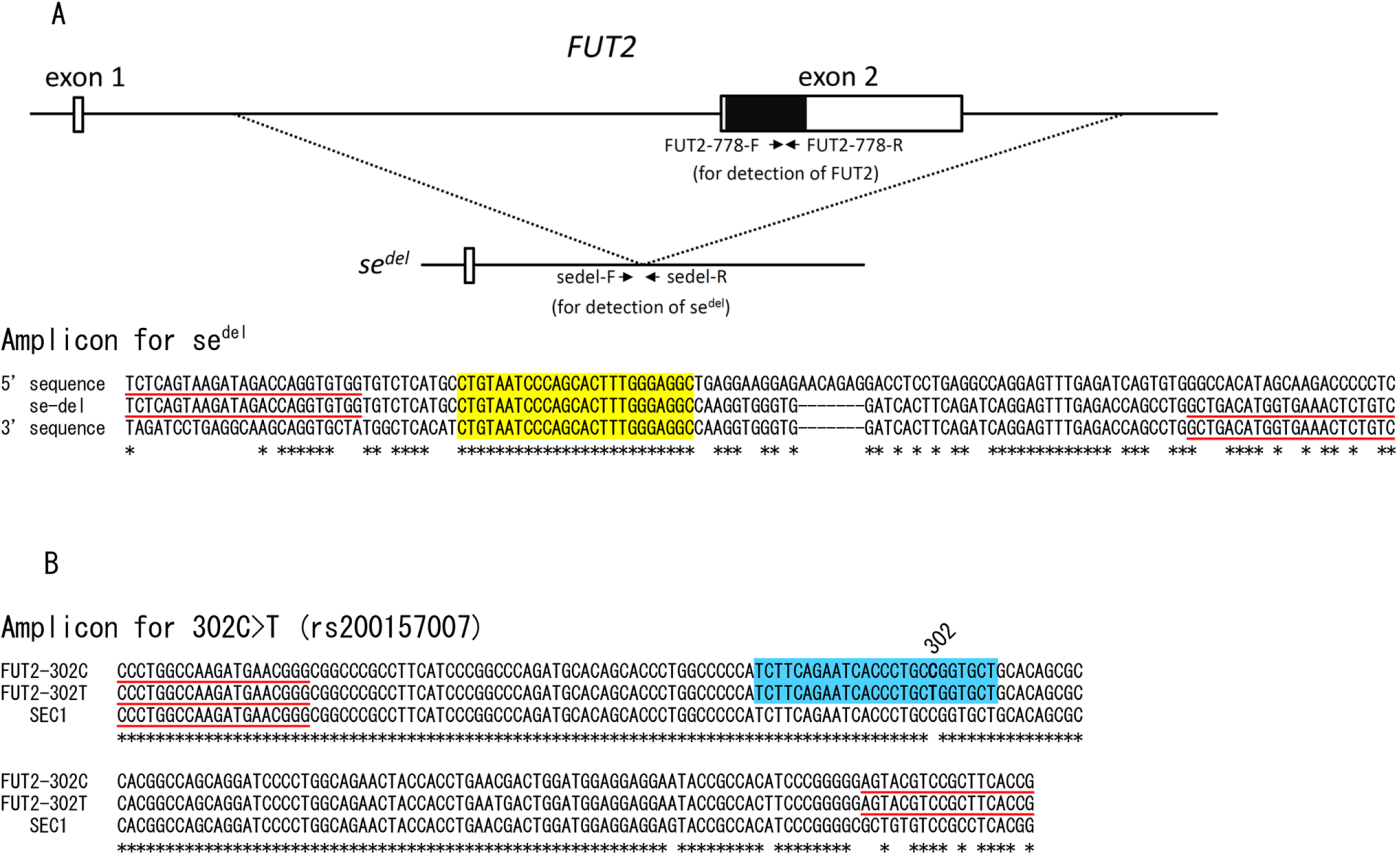
(**A** upper) Gene structure of *FUT2* and *se*^*del*^ and relative position of the deleted region of *se*^*del*^. Two exons of *FUT2* are indicated by white boxes, and the protein coding region in the second exon is indicated by a black box. Positions of PCR primers for duplex real-time PCR to amplify *FUT2* (FUT2-778-F and -R) and *se*^*del*^ (sedel-F and -R) are shown by black arrows. (**A** lower) Alignment of DNA sequences of amplified regions of 127-bp encompassing the *se*^*del*^ junction. DNA sequences of the 5′ breakpoint (5′ sequence), 3′ breakpoint (3′ sequence), and junction region of *se*^*del*^ (se-del) within Alu elements are indicated. Yellow box indicates 25-bp homologous sequence breakpoints at the 5′ and 3′ ends of the deletion. PCR primer sequences (sedel-F and -R) are underlined in red. An asterisk shows an identical nucleotide among the three sequences. (**B**) Alignment of DNA sequences of amplified region of 195-bp encompassing rs200157007 (FUT2-302C: allele of C, FUT2-302T: allele of T at 302C > T), and corresponding regions of *SEC1* are indicated. Blue box indicates the Eprobe sequence. The position of rs200157007 is also indicated as 302. PCR primer sequences are underlined in red. An asterisk shows an identical nucleotide among three sequences.

Five *se* alleles that resulted from copy number variations (CNVs) have also been identified. Four of them (*se*^*del*^, *se*^*del2*^, *se*^*del3*^, *se*^*del4*^) were complete deletions of the coding region, whereas one (*se*^*fus*^) was generated by a homologous recombination between *SEC1* and *FUT2*^9^. Among them, the *se*^*del*^ allele was first identified in subjects of the Indian Bombay phenotype^10^, and, like the *se*^*302*^ allele, is known to be characteristic to South Asians with a frequency of 10–30%^11,12^. This allele was generated by a homologous recombination between two Alu elements that are 10-kb away from each other (Fig. 1A). Alu elements are the most abundant repetitive elements, composing ~ 10% of the human genome. In order to detect *se*^*del*^, we first designed a conventional PCR method to amplify a relatively long fragment (1.8-kb) and then developed a triplex hydrolysis probe (TaqMan) PCR assay to detect CNVs of *FUT2*^13,14^. However, *se*^*del*^, *se*^*del2*^, *se*^*del3*^, and *se*^*del4*^ cannot be discriminated by the triplex TaqMan PCR assay.

Sri Lanka has a diverse ethnic composition and 74% are Sinhalese and 18% are Tamils. We have determined genetic diversity of the *FUT2* previously for the same population in this study and found that *se*^*del*^, *se*^*302*^ and *se*^*428*^ were common *se* alleles as with other South Asian populations. The frequency of *se*^*del*^ was reported to be 28 and 13%, that of *se*^*302*^ was 9.5 and 27% and that of *se*^*428*^ was 9.5 and 22% for Tamils and Sinhalese, respectively^12^.

Recent studies suggested that *FUT2* polymorphism (secretor status) is associated with susceptibility to various infectious diseases, such as norovirus, rotavirus, COVID-19, and several clinical conditions such as Crohn's disease and low plasma vitamin B_12_ levels^15–18^. Large scale replication studies of various populations or independent samples are important for confirmation of these associations. Therefore, accurate and high-throughput genotyping should to be performed. However, common nonsecretor alleles are not shared by different continental populations.

An Eprobe-mediated PCR method (Eprobe-PCR) was recently developed for detection of SNPs. Eprobe is a hybridization-dependent fluorescence probe based on the quenching of two dye moieties in the condition of a single-stranded oligonucleotide and can be applied to sequential quantitative PCR, followed by melting curve analysis in a single reaction tube with a real-time PCR instrument^19^.

The aim of present study was to develop high-throughput methods for detection of *FUT2* polymorphisms applicable to South Asians and to examine the molecular basis of Indian Bombay phenotype in more detail. For this purpose, we developed a duplex real-time PCR melting curve analysis for detection of *se*^*del*^ using a short (127-bp) amplicon together with the *FUT2* coding region using a 65-bp amplicon to determine *se*^*del*^ zygosity in a single tube. We also developed an Eprobe-PCR method for detection of 302C > T of *FUT2* using a 195-bp amplicon*.*

## Results

The Lewis phenotype on red cells of each individual (58 Tamil and 54 Sinhalese) had been determined previously^12^. Le(a − b +) was identified as a secretor and Le(a + b −) as a nonsecretor whereas discrimination of secretors from nonsecretors among 26 Le(a − b –) subjects by phenotyping is impossible. There was no discrepancy between phenotype and genotype determined by Sanger sequencing and denaturing high-performance liquid chromatography (dHPLC) in Lewis-positive subjects^12^. As described previously, we could define that 16 of 26 Le(a − b −) subjects were secretors and ten of them were nonsecretors by genotyping of the *FUT2*^12,20^.

### PCR amplification of *se*^*del*^

First we amplified a PCR product using a set of primers encompassing a 127-bp region of an *se*^*del*^ breakpoint (sedel-F and sedel-R) on a real-time PCR platform (Fig. 1A). Specific amplification of the deletion breakpoint of *se*^*del*^ was confirmed by an amplification signal only from individuals having *se*^*del*^ and direct DNA sequencing of the PCR products of four selected subjects (data not shown).

### Duplex real-time PCR method for detection of *se*^*del*^

**Figure 2:**
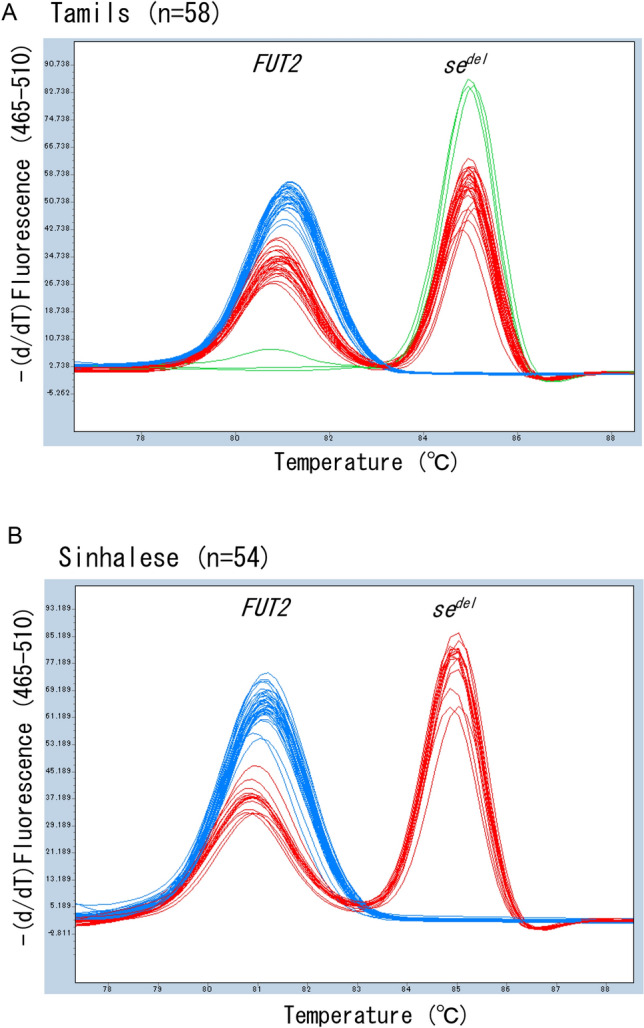
Melting peak profiles of duplex PCR for detection of *se*^*del*^ zygosity. Melting peak profiles obtained for 58 randomly selected Tamils (**A**) and 54 randomly selected Sinhalese (**B**). The individuals with genotypes of +/+ (wild type, blue), +/− (heterozygote of *se*^*del*^, red), and −/− (homozygote of *se*^*del*^, green) were completely separated by melt curve genotyping.

We then designed a duplex real-time PCR to determine the zygosity of *se*^*del*^ in a single tube. In addition to primers for the 127-bp *se*^*del*^-specific amplicon, we added primers for detection of *FUT2* that lacked the *se*^*del*^ allele in a single tube. We performed this on three selected individuals with genotypes of the wild type (+/+), heterozygote of *se*^*del*^ (+/−), and homozygote of *se*^*del*^ (−/−). Since these primers amplified a 65-bp region of the coding region of *FUT2* (751–815 bp), the melting curve analysis of the duplex real-time PCR clearly distinguished the three genotypes from each other (data not shown). The melting temperature (Tm) value of the 127-bp amplicon of *se*^*del*^ was around 85 °C, while that of 65-bp *FUT2* coding region was around 81 °C. The lower limits of discrimination were around 16, 64, and 8 pg of DNA for −/− (homozygote of *se*^*del*^), +/− (heterozygote of *se*^*del*^), and +/+ (wild type), respectively (data not shown). We then applied this method to analyze 58 Tamil and 54 Sinhalese samples and clearly discriminated three genotypes (Fig. 2). However, the results of *se*^*del*^ zygosity of the present method were different from those of the previous conventional PCR assay for one Tamil and two Sinhalese^12^. We reanalyzed these three individuals by conventional PCR. Although we had judged these three individuals to be the +/+ genotype by conventional PCR previously, reanalysis suggested that three individuals were the +/− genotype. Thus the results of *se*^*del*^ zygosity determined by the present method were completely identical to those by the conventional PCR genotyping method, and the numbers of +/+, +/−, and −/− genotypes were 28, 27, and 3 for Tamils and 38, 16, and 0 for Sinhalese. The repeatability was confirmed by two independent assays.

### Genotyping of 302C > T of FUT2 using Eprobe-PCR

**Figure 3:**
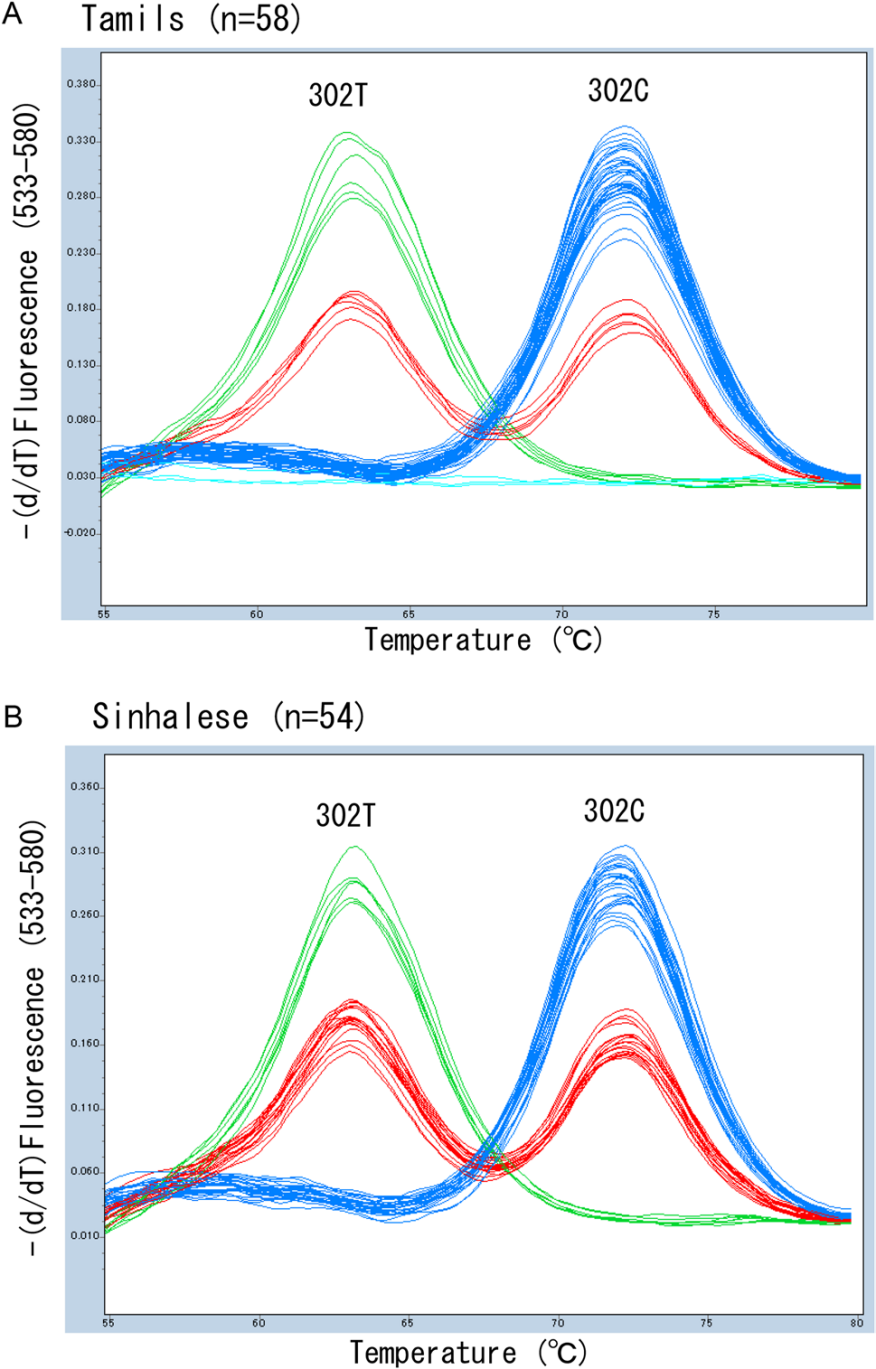
Melting peak profiles of Eprobe-PCR for detection of 302C > T of *FUT2*. Melting peak profiles obtained for 58 Tamils (**A**) and 54 Sinhalese (**B**). The individuals with genotypes of C/C (blue), C/T (red), and T/T (green) at rs200157007 were completely separated by melt curve genotyping. In addition, no amplification signal (and therefore no melting peak) was obtained in three Tamils who were homozygous for *se*^*del*^ (light blue).

Next, we performed melting curve genotyping using a 195-bp amplicon and a 25-bp Eprobe (see Fig. 1B). To amplify *FUT2* specifically, we selected a reverse primer with a seven-base difference from *SEC1* (which is identical to the reverse primer of an unlabeled probe based on high-resolution melt (HRM) analysis for detection of 385A > T of *FUT2*)^21^. Using this method, we clearly distinguished C/C (Tm: around 72 °C), C/T (Tm: around 63 °C and 72 °C), and T/T (Tm: around 63 °C) genotypes from each other (data not shown). We then applied it to 58 Tamils and 54 Sinhalese whose 302C > T genotypes had been determined by Sanger sequencing and dHPLC previously. As shown in Fig. 3, the three genotypes of 302C > T of *FUT2* could be separated clearly, the results were fully in agreement with previous genotyping results, and the numbers of C/C, C/T, and T/T were 43 (including 19 individuals with C/− genotype), 7, and 6 (all 6 individuals were T/− genotype) in Tamils and 28 (including 13 individuals with C/− genotype), 19, and 7 (including 3 individuals with T/− genotype) in Sinhalese. The repeatability was confirmed by two independent assays. In addition, we did not obtain any amplification signal in real-time PCR of three Tamils with the −/− genotype. The results suggested the specific amplification of *FUT2* but not *SEC1*.

## Discussion

We recently developed several HRM-based real-time PCR methods for detection of *se*^*fus*^, *se*^*428*^, and *se*^*385*^ alleles^21–23^. Predominant *se* alleles in several South Asian populations are *se*^*428*^, *se*^*302*^, and *se*^*del*^^12^. In addition to these three *se* alleles, *se*^*385*^ has relatively high frequency in Bangladeshi^11^. Therefore, we should genotype *se*^*428*^, *se*^*302*^, and *se*^*del*^ in many South Asians, whereas *se*^*428*^, *se*^*385*^, *se*^*302*^, and *se*^*del*^ should be genotyped in Bangladeshi for association studies of *FUT2*^8,24^.

Even when we do not consider the deletion allele (*se*^*del*^), the estimation of secretor status is not affected. For example, the *Se*/− genotype is judged as *Se*/*Se* and the *se*/− genotype as *se*/*se*. However, we need to be alert for *se*^*del*^ when targeting a population with high frequency of it. In this study, we did not detect any real-time PCR amplification signal of 302C > T in three Tamils with the −/− genotype (Fig. 3A). The results persuaded us to screen for *se*^*del*^ in South Asians; otherwise, we could not know whether the reason for the lack of an amplification signal of *FUT2* was actually an absence of *FUT2* or another problem, such as degradation of genomic DNA. In addition, it is likely that we overestimated homozygotes. In fact, without considering the results of *se*^*del*^ screening, we misjudged the 302C/*−* genotype of 19 Tamils and 13 Sinhalese as C/C and 302T/*−* genotype of 6 Tamils and 3 Sinhalese as T/T by the Eprobe-PCR assay.

Unfortunately, we overlooked three individuals with the +/− genotype by previous conventional PCR for detection of an 1.8-kb amplicon because of the relatively large size of the PCR product and by simplex PCR without an amplification control^12^. On the other hand, the present duplex real-time PCR melting curve analysis included an amplification control (65-bp *FUT2* coding sequence) that also allowed determination of *se*^*del*^ zygosity in a single tube. Therefore, the present duplex real-time PCR assay for detection of *se*^*del*^ is more reliable and faster than previous conventional PCR methods.

We previously developed a triplex TaqMan PCR assay to detect CNVs of *FUT2*^14^. The advantage of this method is not only detection of known CNVs but the potential to detect novel CNVs. However, it depends largely on both the quality and quantity of DNA to work well and carries a cost in terms of three probes. On the other hand, the real-time PCR melting curve analysis we present here is dedicated to detection of the *se*^*del*^ and unable to detect other CNVs. The present assay is cost-effective, easy to use, straightforward, and not very dependent on both quality and quantity of DNA.

Because a 164-bp sequence surrounding 302C > T is completely identical to that of *SEC1* and this sequence contains another high frequency SNP (about 50% in global populations), 357C > T (rs281377, synonymous SNP), it was difficult to select appropriate primers for short amplicon HRM to detect 302C > T of *FUT2*. For this reason, in this study, we employed Eprobe-PCR instead of HRM analysis for detection of 302C > T of *FUT2*. Compared with HRM analysis using a short amplicon, Eprobe-PCR needs a labeled probe, and is therefore more expensive. However, the HRM method is based on detection of subtle differences of the melting curve and melting temperature of PCR amplicons, whereas the Eprobe-PCR method is based on detection of relatively large differences of the melting curve and melting temperature of a short probe sequence. Therefore, a probe-based melting curve analysis seems to be one of the most specific and sensitive methods to detect SNPs^25^. In fact, the Tm values of the wild-type (around 72 °C) and that of the mutant (around 63 °C) were quite different (around 9 °C). This significant difference made a clear distinction of the three genotypes of C/C, C/T, and T/T possible. Thus we believe that the present Eprobe-PCR for detection 302C > T of *FUT2* is quite useful and reliable.

In conclusion, the present two protocols seem to be a reliable and high throughput method for detection of *se*^*del*^ and *se*^*302*^ in South Asian subjects and for examination of genetic basis of Indian Bombay phenotype in more detail.

## Materials and methods

### Statements and DNA samples

All methods were carried out in accordance with relevant guidelines and regulations. DNA samples from 58 Tamil and 54 Sinhalese in Sri Lanka whose *FUT2* genotypes had been already determined were used^12^. Genomic DNA was extracted from frozen whole blood using Gentra Puregene Blood Kit (Qiagen, Tokyo, Japan). The oral informed consent was obtained and the DNA samples were taken from participants in 2002. The statement for oral informed consent approved by ethical committee of Kurume University in 2002. However, present study protocol was approved by the ethical committee of Kurume University School of Medicine in 2017 using existing and already anonymized DNA samples (Bioethics approval No. 342).

### Duplex real-time PCR melting curve analysis for detection of *se*^*del*^

Since both the 5′ and 3′ deletion breakpoints of *se*^*del*^ are located within Alu-repetitive elements^13^, the primers were carefully designed to amplify only a recombination allele (*se*^*del*^) but not other Alu-elements using Primer 3 (https://bioinfo.ut.ee/primer3-0.4.0/)^26^ and a BLAST search (https://blast.ncbi.nlm.nih.gov/Blast.cgi) (Fig. 1A). For amplification of the 127-bp amplicon of *se*^*del*^ encompassing a homologous sequence of 25 bp at each breakpoint, we selected a sedel-F primer (5′-TCTCAGTAAGATAGACCAGGTGTGG-3′, 35–11 bp upstream from the 5′ end of the 25-bp homologous sequence) and a sedel-R primer (5′-GACAGAGTTTCACCATGTCAGC-3′, 46–67 bp downstream from the 3′ end of the 25-bp homologous sequence) from several candidates. For duplex PCR to determine the zygosity of *se*^*del*^, we added primers which were recently designed for HRM analysis (FUT2-778-F: 5′-TTTGCTGGCGATGGCATT-3′ and FUT2-778-R: 5′-TGGTTACACTGTGTGAGTAGAGCAA-3′) to detect 778C > del (P260Lfs*16, rs1799761) as *FUT2*-specific primers^23^. These primers amplified the 751–815 bp coding region of *FUT2* which lacked the *se*^*del*^ allele (Fig. 1A). The primers were synthesized by Eurofins Genomics (Tokyo, Japan). The 10 µL PCR reaction contained 1–10 ng genomic DNA, 5 µL of LightCycler 480 HRM Master mix (Roche Diagnostics, Mannheim, Germany), 2.5 mM MgCl_2_, 125 nM of sedel-F and sedel-R primers, and 187.5 nM of FUT2-778-F and FUT2-778-R primers. Touchdown PCR was performed on a LightCycler 480 instrument II (Roche Diagnostics). The thermal profile was as follows: one cycle at 95 °C for 10 min, denaturation at 95 °C for 10 s, annealing at 64 °C for 20 s with touchdown every 0.5 °C, and extension at 72 °C for 20 s for 12 cycles, and then 95 °C for 10 s, 58 °C for 20 s, and 72 °C for 20 s for 33 cycles. The fluorescence data for monitoring real-time PCR amplification were collected at the end of the annealing step of each cycle using a filter (465–510 nm). The products were heated to 95 °C for 1 min, rapidly cooled to 40 °C for 1 min, and then the fluorescence data for melting curve analysis were collected over the range from 75 to 90 °C, increasing at 0.02 °C/s with 25 acquisitions/s. The melting curve genotypes were automatically clustered into separate groups by LightCycler 480 Gene Scanning Software (Roche Diagnostics).

### Sanger sequencing of PCR products of *se*^*del*^

PCR amplification was performed using sedel-F and sedel-R primers and LightCycler 480 HRM Master mix as describe above. Direct sequencing of the PCR amplicons of *se*^*del*^ was performed using each PCR primer as a sequence primer^4^.

### Eprobe-PCR for detection of 302C > T of *FUT2*

Nucleotide positions of the *SEC1* and *FUT2* genes are numbered as described previously^27^. For PCR amplification surrounding 302C > T, Primer3 (https://bioinfo.ut.ee/primer3-0.4.0/) was also used to design primers for specific amplification of *FUT2*. In addition, Edesign software ver.2.00 (http://www.dnaform.com/edesign2/) provided by K.K. DNAFORM (Yokohama, Japan) was used to design an Eprobe for detection of 302C > T of *FUT2*. For Eprobe-PCR, we used a FUT2-302-F primer (5′-CCCTGGCCAAGATGAACGG-3′, 218–236 bp of *FUT2* and identical with the corresponding *SEC1* sequence, Fig. 1B underlined) a FUT2-302-R primer (5′-CGGTGAAGCGGACGTACT-3′, 395–412 bp of *FUT2*, a 7-base difference from *SEC1*, Fig. 1B underlined) and Eprobe (5′-TCTTCAGAAUCACCCTGCCGGTGCT-3′-AmC3; U indicates the position of the modified T by thiazole orange, 284–308 bp of *FUT2*). The Eprobe was blocked on the 3′ end (3′-amino-modifier C3) to prevent extension during PCR. The primers were synthesized by Eurofins Genomics, and the Eprobe was synthesized by K.K. DNAFORM. We performed real-time PCR and melting curve analysis using a LightCycler 480 Instrument II. Asymmetric PCR amplification was performed in 10 μL reaction mixture including 1–10 ng of genomic DNA, 5 µL of E-Taq 2 × PCR Mix (K.K. DNAFORM), 50 nM of FUT2-302-F primer, 250 nM of FUT2-302-R primer, and 250 nM of the Eprobe. The thermal profile was as follows: one cycle at 95 °C for 30 s, followed by 50 cycles with denaturation at 95 °C for 15 s, annealing at 58 °C for 30 s, and extension at 72 °C for 15 s. The fluorescence data for monitoring real-time PCR amplification were collected the end of the annealing step of each cyle using a filter (533–580 nm). The products were heated to 95 °C for 1 min, rapidly cooled to 45 °C for 1 min, and fluorescence data for melting curve analysis were collected over the range from 50 to 80 °C. The melting curve genotypes were automatically clustered into separate groups by LightCycler 480 Gene Scanning Software (Roche Diagnostics).
